# PaCMAP-embedded convolutional neural network for multi-omics data integration

**DOI:** 10.1016/j.heliyon.2023.e23195

**Published:** 2023-12-05

**Authors:** Hazem Qattous, Mohammad Azzeh, Rahmeh Ibrahim, Ibrahim Abed Al-Ghafer, Mohammad Al Sorkhy, Abedalrhman Alkhateeb

**Affiliations:** a Software Engineering Department, Princess Sumaya University for Technology, Amman P.O. Box 1438, Jordan; b Data Science Department, Princess Sumaya University for Technology, Amman P.O. Box 1438, Jordan; c Computer Science Department, Princess Sumaya University for Technology, Amman P.O. Box 1438, Jordan; d Heritage College of Osteopathic medicine, Ohio University, Cleveland, OH 44122, USA; e Computer Science Department, Lakehead University, 955 Oliver Rd, Thunder Bay, ON P7B 5E1, Ontario, Canada

**Keywords:** Multi-omics data integration, Embedding techniques, PaCMAP, Convolutional neural network

## Abstract

**Aims:**

The multi-omics data integration has emerged as a prominent avenue within the healthcare industry, presenting substantial potential for enhancing predictive models. The main motivation behind this study stems from the imperative need to advance prognostic methodologies in cancer diagnosis, an area where precision is pivotal for effective clinical decision-making. In this context, the present study introduces an innovative methodology that integrates copy number alteration (CNA), DNA methylation, and gene expression data.

**Methods:**

The three omics data were successfully merged into a two-dimensional (2D) map using the PaCMAP dimensionality reduction technique. Utilizing the RGB coloring scheme, a visual representation of the integration was produced utilizing the values of the three omics of each sample. Then, the colored 2D maps were fed into a convolutional neural network (CNN) to forecast the Gleason score.

**Results:**

Our proposed model outperforms the cutting-edge i-SOM-GSN model by integrating multi-omics data and the CNN architecture with an accuracy of 98.89, and AUC of 0.9996.

**Conclusion:**

This study demonstrates the effectiveness of multi-omics data integration in predicting health outcomes. The proposed methodology, combining PaCMAP for dimensionality reduction, RGB coloring for visualization, and CNN for prediction, offers a comprehensive framework for integrating heterogeneous omics data and improving predictive accuracy. These findings contribute to the advancement of personalized medicine and have the potential to aid in clinical decision-making for prostate cancer patients.

## 1 Introduction

The advancement of biomedical technologies has enabled large-scale data generation across different omics platforms. These technologies have enabled researchers to measure various molecular features of biological systems at a high-throughput and high-resolution level, allowing for the generation of comprehensive and complex datasets. Integrating these multiple sources of biological data leads to a better understanding of complex diseases, including cancer [1]; however, the challenge is integrating the generated heterogeneous data in the same machine-learning model for classification. The technique of concatenating data from multiple data sources may struggle to find the best approach for combining multiple matrices with different scales in a biologically meaningful way [2]. In this model, we utilize the PaCMAP dimensionality reduction technique to embed the various sources of omics in a CNN prediction model.

Many researchers proposed models to extract discriminative features from multi-sources of data [3] [4] [5] [6]. While Razzaghi et al. applied a multimodal feature encoder to extract features from imagery data [3], Vasighizaker et al. used dimensionality reduction (DR) technique to extract multi-omics features from single-cell data [4]. In [5] [6], the models merged different data sources for optimization purposes. Rafiei et al. proposed a model named DeepTraSynergy that combines protein-protein interactions (PPIs), cell–target interaction, and drug sequences as input. It also predicts the drug–target interaction, its toxic effect, and drug combination synergy as different tasks. DeepTraSynergy built a specific type of network for each data source, including Drug feature extraction network, Compound–protein interaction network, and Synergy network [7]. DR techniques are essential in data visualization because they make it easier to understand and analyze high-dimensional data by making it less complicated. Self-organizing maps (SOM), uniform manifold approximation and projection (UMAP), and t-distributed stochastic neighbor embedding (t-SNE) are all algorithms that have been made for this purpose. Each of these algorithms has its own strengths and limitations, and the optimal choice depends on the nature of the data and the problem at hand [8].

SOM is a neural network that employs unsupervised learning techniques to adapt weights based on the input data, resulting in a more interpretable visualization of multidimensional data for human comprehension [9]. The key benefits of SOM data mapping are that it is simply interpreted and capable of organizing big, complex datasets. On the other hand, the key disadvantages of this DR approach include difficulty deciding the input weights to utilize. It takes adequate and required data to generate meaningful clusters. Large datasets can be computationally expensive, and creating fine mapping is problematic when categories are unique within the map [10].

The second DR technique, t-SNE, is a well-known dimensionality reduction technique for visualizing high-dimensional data [11]. Unlike other dimensionality reduction approaches, t-SNE does not focus on preserving the linear relationships among the data points. Instead, it emphasizes retaining the local structure of the data points [12]. As a result, t-SNE is an excellent choice for visualizing complex and non-linear data. It is also easy to use and implement, with a wide range of implementations available in various programming languages, and is highly customizable, allowing users to adjust different parameters to obtain the best results from the data. t-SNE, on the other hand, has some limitations, including slow computation time, which makes it unsuitable for very large datasets, the inability to represent very large datasets and loss of large-scale information meaningfully, and the inability to guarantee the global structure of the data, which makes it less suitable for some applications.

The UMAP DR technique appears competitive with t-SNE as a robust technique for visualization quality in DR. UMAP also preserves more of the global structure while retaining the local structure [13]. Furthermore, the topological basis of UMAP allows it to scale far larger datasets, faster processing speed, and better visualization than t-SNE. UMAP also has no computational constraints on the embedding dimension, making it suitable for dimension reduction. Also, UMAP can handle both linear and non-linear structures. The main disadvantages of UMAP are that it can be sensitive to hyper-parameter selection and that the visualization may need to be more obvious for non-linear data [14].

In this study, we incorporate a recent DR technique known as “pairwise controlled manifold approximation (PaCMAP)”, which is a dimensionality reduction method that optimizes low-dimensional embedding by utilizing three kinds of point pairs: neighbor pairs, mid-near pairs, and farther pairs. This novel technique improves on the global versus local trade-off, performs well without parameter tuning, is substantially quicker than other algorithms, achieving a speedup of more than 1.5 times faster than other methods for most datasets, and is simple to use. The algorithm's hyper-parameters are also highly intuitive and can be applied to effortlessly transition from a focus on local to a global structure. Therefore, PaCMAP maintains the global structure without surrendering the local structure or relying on initialization [14].

## 2 Related work

SOMs are single-layer neural networks [9]. In each node, weights have been taught to approximate the expression values of analyses based on a set of linked observations. Each observation is put in the best-fitting unit or node on the grid and then changed to reflect the features seen. This mapping method is utilized for training purposes. After training, each unit becomes the center of its cluster. The user must define the grid size in SOM. The user must also supply the neighborhood size, which is the extent of a node's sphere of influence on its neighbors, and the learning rate, which is the rate at which node weights update in each algorithm iteration [9]. There are two specific ways to evaluate SOM results: topographic accuracy [15] and map embedding accuracy [16].

In a paper [17], a deep learning-based system is proposed and used to aggregate data from many measurements to forecast illness stages. The “iSOM-GSN” method uses SOMs to map higher-dimensional multi-omics data onto a 2D grid and use gene expression data to make a Gene Similarity Network (GSN). At the same time, a SOM and a CNN are used to do data integration on large amounts of high-dimensional cancer genomics data. They have also devised a way to use more types of multi-omic data and predict clinical or disease states, such as where a tumor will grow, how long it will live, or its sub-types. Because SOMs learn by competing with each other, the suggested method can also be considered as an unsupervised clustering algorithm. This model was used to predict prostate and breast cancer, and the prediction accuracy was in the range of 94–98% in both cases with only 14 input genes.

In a study [18], the authors employed SOMs to examine high-dimensional genomics datasets of gene expression and chromatin status during the development of the Xenopus tropicalis mesendoderm. They employed morpholino, wild-type, and geographical data to assess the obtained gene expression data and classify genes into developmental period-specific clusters while not knowing when the time points were taken. In an attempt to capture similar regulation, the SOM additionally classified the genes depending on the effect that various morpholinos had on them. Last but not least, the groups had evident functional and/or developmental differences, indicating that they may be co-regulated. Several well-known co-binding/co-regulation connections from Xenopus or other vertebrates were re-captured by the SOM analysis of the ChIP-seq and ATAC-seq data as meta-clustering.

Additionally, in paper [19], a deep learning model that enhances the iSOM-GSN model was introduced based on Clust 5's integration of multi-omics data. Using an integration layer, the model was updated with the predictions from the three CNNs. All of the omic data sets were used to train their model, which then employed CNNs to find one-dimensional sample vectors.

Another use for SOM is shown in the paper [4], where the authors suggested a deep learning method for classifying cell types from single-cell RNA-seq data. Using a SOM and a CNN, the suggested method does dimensionality reduction, feature selection, and classification simultaneously. They found a new way to represent cells using only 13 genes in a two-dimensional space using the SOM learning method. They did this by making a template with the most useful genes. Then, with an accuracy rate of 98%, they identified populations of various cell types in the human pancreas on the test dataset. In the study [20], the authors used SOMs to illustrate the relationships and interactions between the data on DNA methylation and gene expression in gliomas. As a result, they found several modes that show how each mode causes epigenetic modification that causes glioma. Two modes, for instance, control hypermethylation when specific genes are expressed. However, since this information reveals molecular sub-types or modes concerning their epigenetic behavior, it cannot be used to categorize patients or samples.

Distributed stochastic neighbor embedding is another method for multi-omics data dimensionality reduction (t-SNE). The authors of this model [21], first used t-SNE to create a GSN map for each omic, which is then merged into the residual neural network (ResNet) classification model. This study aimed to identify multi-omics genetic markers associated with breast cancer survival prognosis and prediction. The authors evaluated this model and put it up against many high-dimensional embedding techniques and neural network configurations. The suggested model performed an accuracy of 98.48%. On the other hand, the analysis's lack of additional clinical features, such as race, age, and therapeutic response, was the main limitation of this study.

The authors of [22] presented how CNN and a gene similarity network (GSN) based on UMAP and CNNs can be used to integrate multi-omics data. UMAP is used to put copy number alteration (CNA), DNA methylation, and gene expression into a lower dimension, which makes two-dimensional RGB images. The authors in this study built the GSN using gene expression and then combined it with other omics data for improved prediction. They also used CNNs to predict the stage of tumors in people with breast cancer and the Gleason scores of people with prostate cancer. In the initial step of this procedure, a gene similarity network (GSN) on gene expression omics is created using UMAP. This provides a two-dimensional map and a feature template for the high-dimensional gene expression omics. After the model has been updated with all of the omics data, each sample is then shown as a colored image with all of the data filled in. The classification of these images is then forwarded to CNN. The model performs near perfection. While UMAP is well tested on some biological and ecological data, it struggled in embedding some other types of data, which makes it a less candidate for integrating various types of omics in the future [23].

## 3 Materials and methods

### 3.1 Dataset

This study employed the proposed model to analyze the TCGA Prostate Adenocarcinoma (PRCA) dataset [24], which utilizes Gleason scores for prostate cancer aggressiveness classification. The dataset encompasses three omics: CNA, DNA methylation, and gene expression. The dataset contains 499 total samples, split into three classes based on Gleason scores 4+3, 3+4, and the combination of 4+5 and 5+4 is considered a single class due to the small number of samples available for these advanced scores. The number of samples was reduced to 387 because we only selected samples that contained all three omics.

### 3.2 Pre-processing

The gene expression features were initially filtered to eliminate any with a variance of less than 0.2%. This resulted in a drop in the number of gene expression features from around 39,000 to around 16,000. Genes not listed in HUGO format were removed after normalizing all three omics data on an average scale. The MutSigCV algorithm [25] was used as the final step to significantly separate the mutated genes; it determines the False discovery rate (FDR), and genes with FDR≤0.1 were found to have undergone significant mutations. As a result, 14 mutated genes were chosen for this study from the MutSigCV output.

### 3.3 Proposed method

**Figure 1:**
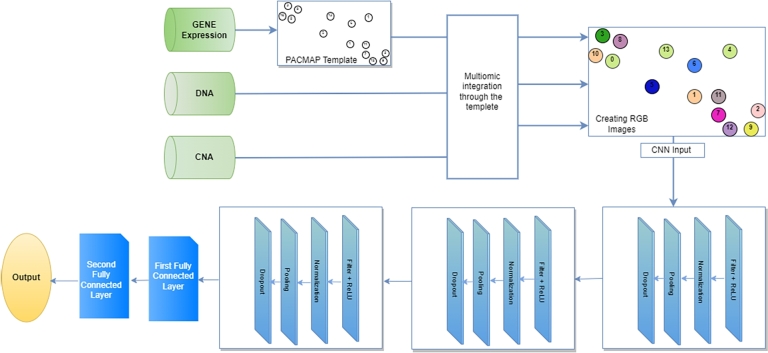
A Schematic view of the proposed workflow for integrating the three omics based on PaCMAP embedding technique into CNN.

Fig. 1 depicts the procedure for our method. On the high-dimensional gene expression omics, it first builds a GSN with PaCMAP to transform it into a two-dimensional map and create a feature template. The template then aggregates all of the omics data and renders each sample as a colored picture with all of the omics data filled in. These pictures are then sent to CNN for classification.

#### 3.3.1 Pairwise Controlled Manifold Approximation Projection (PaCMAP)

PaCMAP is a dimensionality reduction method that may be used for visualization and maintains the local and global structure of the data in the original space. PaCMAP uses three distinct point pairings to maximize low-dimensional embedding: neighbor, mid-near, and further pairs.

Previous dimensionality reduction techniques (such as t-SNE and UMAP) focus on either local structure or global structure, but not both, despite carefully tuning the parameter in their algorithms that control the balance between global and local structure, which primarily adjusts the number of considered neighbors. Instead of considering additional neighbors to draw to maintain the local structure, PaCMAP dynamically employs a particular set of mid-near pairs to capture the global structure and then improve the local structure.

Based on the above-implemented algorithm, PaCMAP consists of three main steps: construction, initialization of the solution, and iterative optimization using a custom gradient descent algorithm.

• *Graph construction* PaCMAP employs edges as graph building blocks. This DR distinguishes between neighbor pairs, mid-near pairs, and further pairs of edges. The first group is made up of each observation's number of closest neighbors in the high-dimensional space. The metric scaled distance is defined as appears in equation (1):
  (1) $$d_{ij}^{2,select}=\frac{{\left\|Xi-Xj\right\|}^{2}}{\sigma ij}\;and\;\sigma ij=\sigma i\;\sigma j,$$
  where *σi* is the average separation between *i* and its fourth to sixth closest Euclidean neighbors. The scaling is done to consider the possibility that neighborhoods in various regions of the feature space could have very different magnitudes. When choosing neighbors, in this case, the scaled distances $d_{ij}^{2,select}$ are only used; they are not used for optimization.
  The second group comprises a number of mid-near pairs, which are chosen randomly. In addition, the third group is made up of additional points that were randomly chosen from each observation.
  For each kind of pair, PaCMAP employs three different loss functions in equation (2):
  (2) $$Loss_{NB}=\frac{{\overset{˜}{d}}_{ij}}{10+{\overset{˜}{d}}_{ij}}\;Loss_{MN}=\frac{{\overset{˜}{d}}_{ik}}{10000+{\overset{˜}{d}}_{ik}},Loss_{FP}=\frac{1}{1+{\overset{˜}{d}}_{il}}.$$
  Where ${\overset{˜}{d}}_{ab}={\left\|{\text{y}}_{a}-{\text{y}}_{b}\right\|}^{2}+1$. The coefficients wNB, wMN, and wF P, which combine to find the total loss, are added as additional weightings for the pairs.
• *Initialization of PaCMAP*
  Although the initialization method has little effect on the results of PaCMAP, the Principal component analysis (PCA) DR is still used to actually reduce the running time.
• *Dynamic Optimization*
  Three phases make up the optimization process, each of which is intended to prevent local optima. The objective of the initial placement of embedded points in the first phase is to improve it in a way that keeps the local and global structures, but primarily the global structure. The mid-near pairs are heavily weighted to achieve this. The second phase's objective is to improve local structure while keeping the overall structure established in the initial phase by giving mid-near couples a minimal weight (but not zero). The third phase focuses on reducing the weight of mid-near pairs and neighbors, which aims to enhance the local structure.

#### 3.3.2 Omics integration and gene-similarity networks

**Figure 2:**
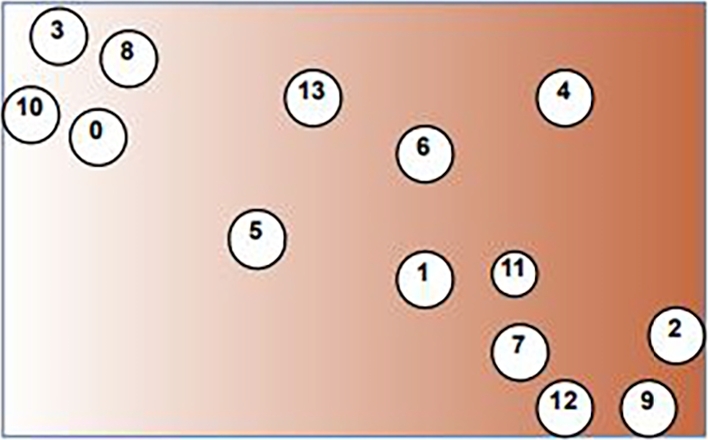
The GSN map was built by applying PaCMAP to gene expression omic.

**Figure 3:**
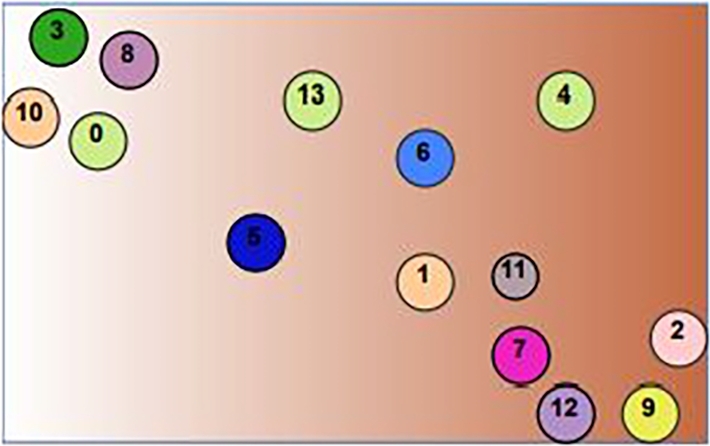
The GSN map after coloring it by integrating the three omics values into the RGB system.

We use PaCMAP for the gene expression omics, which generates the GSN and displays the genes on a two-dimensional map. The two-dimensional map shows the relationships between related genes as well as how similar or dissimilar the genes are arranged. Following the design of the two-dimensional map, all omics data is integrated. The integration is carried out as shown in Fig. 2 by constructing a circular zone with a predetermined radius around the gene sites and then filling those zones with various colors depending on the kind of omics, as shown in Fig. 3. A data sample won't contribute to the RGB palette's color if it is only a particular distance from a gene point. Gene expression is supported by the red color (R), DNA methylation by the green color (G), and CNA by the blue color (B).

## 4 The prediction model

Convolutional neural networks, also referred to as CNNs or ConvNets, are a subclass of deep learning that is responsible for processing data with a grid layout, like images [26] [27].

CNNs contain three types of layers: a convolution layer, a pooling layer, and a fully connected layer. The convolution and pooling layers perform feature extraction using different types of filters, whereas the fully connected layer, maps the extracted features into the final output for the classification process. A convolution layer in CNN is composed of several operations, such as convolution, and a specialized type of activation or transfer function [28] [27] [29].

The following describes the structure of our CNN: The first convolutional layer and the second convolutional layer are the same, which contain 32 convolutional filters with filter size equal to 3×3, a max pooling layer of size equal to 2×2 and 1×1 stride step, a normalized layer, and a dropout layer of 20% rate. In third convolutional layer consists of 32 convolutions with a filter size equal to 3×3, a max pooling layer of size equal to 2×2 size and 2×2 stride steps, a normalized layer, and a dropout layer of 50% rate.

In three convolutional layers, we used a rectified linear unit (ReLU) activation function, and the purpose of using the normalized layer and dropout layer was to overcome the over-fitting issue. The detailed steps for our proposed method are shown in Algorithm 1.

**Algorithm 1:**
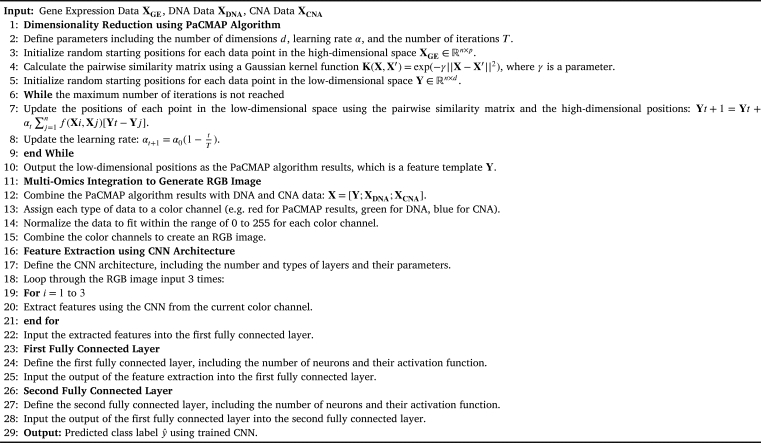
Multi-Omics Integration using PaCMAP.

Stochastic gradient descent (SGD) is widely used in optimizing the learning process [30]. In this model, we adopted Adam optimizer is a faster extension of the SGD [31] in the training to minimize the loss function.

## 5 Experiments and results

We used the prostate cancer dataset in this experiment to test our suggested model. We used grid search to enhance the model's performance and found that the best accuracy was achieved with a learning rate of 0.05 and 80 epochs. A training set comprising 70% of the datasets and a testing set, 30%, were separated. In order to compare the performance of our suggested approach with that of the iSOM-GSN model, we also ran it on the dataset using the default value. As indicated in Table 1, our suggested strategy produced remarkable results in the testing set, achieving over 99% accuracy across all evaluation metrics. To assess the performance of our model, we employed the evaluation metrics shown in equations ((3), (4), (5), and (6)) as follows:

(3) $$\text{Recall}=\frac{\text{TP}}{\text{TP}+\text{FN}}$$

(4) $$\text{Precision}=\frac{\text{TP}}{\text{TP}+\text{FP}}$$

(5) $$\text{Accuracy}=\frac{\text{TP}+\text{TN}}{\text{TP}+\text{TN}+\text{FP}+\text{FN}}$$

(6) $$\text{F1-measure}=\frac{2⁎(\text{precision}⁎\text{recall})}{\text{precision}+\text{recall}}$$

**Table 1:** Performance assessment of the suggested model and the iSOM-GSN.

Performance measurements	The PRCA Dataset	
	Proposed model	iSOM-GSN
Accuracy	98.89%	97.89%
Precision	98.89%	98.82%
Recall	98.89%	98.72%
F1-measure	98.89%	98.71%
AUC	0.9996	0.9913

The True Positive (TP) indicator shows how many correctly predicted positive courses there were when the actual class was positive, while the True Negative (TN) indicator shows how many correctly predicted negative classes there were when the actual class was negative. False Positives (FP) measure the proportion of falsely predicted positive classes when the actual class is negative, and False Negatives (FN) measure the proportion of falsely predicted negative classes when the actual class is positive.

**Figure 4:**
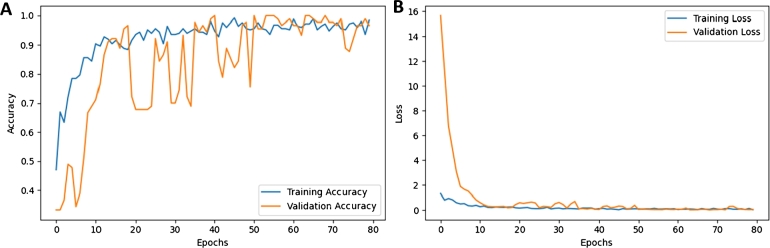
(A) Training and validation accuracy for the proposed model. (B) Loss results for the proposed model.

**Figure 5:**
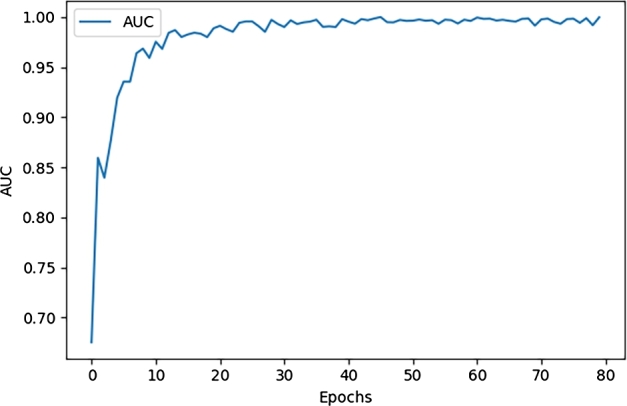
AUC Results for prostate cancer dataset.

Table 1 displays the evaluation metrics of the proposed model and the iSOM-GSN model. The proposed model achieved an accuracy, precision, recall, and F1-measure of 98.89% outperforming iSOM-GSN model. Additionally, it achieved an AUC of 0.9996 compared to 0.9913 for iSOM-GSN. Fig. 4 shows the training and validation accuracy and loss for the Proposed model. Also, Fig. 5 presents the area under the curve (AUC) for running the proposed model with various numbers of epochs.

## 6 Discussion

Various heterogeneous omics data need to transform into the latent variables that represent major underlying biological processes in each omic [32]. In the input classification layer, directly concatenating these different in-nature data with different scales may lead to bias or inconsistent prediction model performance [22]. Earlier models in transforming multi-omics data into latent variables used PCA as an embedding technique [33], which implicitly assumes the linear relationships among the features. With the earlier mentioned disadvantages of current embedding techniques in the literature, we proposed PaCMAP DR as an embedding method to transform the various omics into latent space before merging them into the classification model.

The generated map reduces the 16-dimensional features into a visualized 2-dimensional picture. The visual space represents the relationships between the extracted 16 genes in the x-axis and y-axis style. The value from each omic of the sample contributes to the map by coloring a channel in the RGB system. The result section shows how this model outperformed iSOM-GSN. Fig. 4 shows how the accuracy of the training and validation fluctuated over running the model on various numbers of epochs, the loss between them was relatively small.

## 7 Conclusion

In conclusion, integrating multi-omics data has emerged as a promising approach for enhancing prediction models in the healthcare industry. This study presents a novel methodology that combines copy number alteration (CNA), DNA methylation, and gene expression data to predict the malignancy Gleason score for individuals with prostate cancer. A visually informative representation of the integrated data was generated by successfully merging the three omics data into a two-dimensional (2D) map using the PaCMAP dimensionality reduction technique and utilizing the RGB coloring scheme. This colored 2D map was fed into a CNN to predict the Gleason score class.

The performance measurements of the proposed model surpassed those of the state-of-the-art i-SOM-GSN model by leveraging the integration of multi-omics data and the CNN architecture. The model highlights the efficacy of multi-omics data integration in predicting health outcomes. The proposed methodology, which combines PaCMAP for DR, RGB coloring for visualization, and CNN for prediction, provides a comprehensive framework for integrating heterogeneous omics data and improving predictive accuracy. The future work will embed sequence data and sparse data, including time series and mutation frequency, respectively, to validate the model to more data types. The source code is available at https://github.com/dtabed/PacMapOmics.

## CRediT authorship contribution statement

**Hazem Qattous:** Writing – review & editing, Writing – original draft, Methodology, Funding acquisition, Formal analysis, Conceptualization. **Mohammad Azzeh:** Writing – original draft, Validation, Software, Resources, Formal analysis, Data curation, Conceptualization. **Rahmeh Ibrahim:** Writing – review & editing, Writing – original draft, Methodology, Formal analysis, Conceptualization. **Ibrahim Abed Al-Ghafer:** Writing – original draft, Methodology, Conceptualization. **Mohammad Al Sorkhy:** Writing – original draft, Validation, Investigation. **Abedalrhman Alkhateeb:** Writing – review & editing, Writing – original draft, Supervision, Methodology, Investigation, Funding acquisition, Conceptualization.

## Declaration of Competing Interest

The authors declare the following financial interests/personal relationships which may be considered as potential competing interests:

Hazem Qattous reports financial support was provided by Hashemite Kingdom of Jordan Scientific Research Support Fund with grant number (ICT/1/16/2022). Abedalrhman Alkhateeb reports financial support was provided by Hashemite Kingdom of Jordan Scientific Research Support Fund with grant number (ICT/1/16/2022). If there are other authors, they declare that they have no known competing financial interests or personal relationships that could have appeared to influence the work reported in this paper.

## Data Availability

Data will be made available on request.
